# *CDCA7* Promotes Proliferation and Suppresses Apoptosis in Gastric Cancer via HELLS-Mediated Chromatin Remodeling

**DOI:** 10.32604/or.2026.076051

**Published:** 2026-04-22

**Authors:** Jun Jiang, Yi-Ran Li, Xiaoting Wang, Jian Li, Fangzhou Ye, Jiayi Wang, Huanqing Li, Li Feng

**Affiliations:** 1 Endoscopy Center, Minhang Hospital, Fudan University, No. 170 Xinsong Road, Shanghai, China; 2 Department of Ultrasound, Eastern Hepatobiliary Surgery Hospital, The Third Affiliated Hospital of Naval Medical University, Shanghai, China; 3 Qibao Community Health Service Center, 94 Fuqiang Street, Shanghai, China

**Keywords:** *CDCA7*, *HELLS*, chromatin remodeling, gastric cancer, proliferation, apoptosis

## Abstract

**Background:**

In various tumor types, cell division cycle-associated 7 (*CDCA7*) is involved in chromatin remodeling and DNA methylation. However, its biological functions and regulatory mechanisms in gastric cancer (GC) remain unknown. This investigation intended to identify the function of *CDCA7* in GC progression and elucidate its epigenetic regulatory mechanisms.

**Methods:**

Differentially expressed genes (DEGs) were detected from the GSE19826, TCGA-GC, and GSE56807 datasets. Networks of protein-protein interactions (PPI) and hub genes were discovered by the DMNC and Clustering Coefficient algorithms. Receiver operating characteristic (ROC) analysis and expression profiling were undertaken to determine diagnostic performance. *In vitro* assays, including CCK-8 assays, clonogenic assays, flow cytometry, dot blots, co-immunoprecipitation (Co-IP), chromatin immunoprecipitation (ChIP), and Western blots, were applied to evaluate the role of *CDCA7* and its interaction with helicase, lymphoid-specific (*HELLS*).

**Results:**

169 overlapping genes were discovered, enriched in Cell adhesion molecules and ECM-receptor interaction. *CDCA7* is highly expressed in GC and has high clinical diagnostic value. Knockdown of *CDCA7* causes apoptosis and suppresses GC cell invasion, migration, and proliferation. Mechanistically, CDCA7 physically interacts with HELLS and promotes HELLS recruitment to chromatin. Knockdown of *CDCA7* reduces global 5 hmC/5 mC levels and histone methylation (H3K9me3 and H4K20me3), while *HELLS* overexpression partially reverses these effects. Functionally, *HELLS* overexpression also partially reverses the antiproliferative and proapoptotic effects of *CDCA7* knockdown.

**Conclusion:**

*CDCA7* promotes GC progression by interacting with *HELLS* to regulate DNA methylation and chromatin stability, suggesting that the *CDCA7*-*HELLS* axis may serve as a potential diagnostic biomarker and therapeutic target for GC.

## 1 Introduction

Cancer remains one of the leading causes of morbidity and mortality worldwide, and its development is driven by a complex interplay of genetic alterations, epigenetic dysregulation, and environmental factors. Over the past decades, cancer treatment strategies have evolved from conventional surgery and cytotoxic chemotherapy to precision medicine approaches, including targeted therapy and immunotherapy; however, treatment resistance and tumor heterogeneity continue to pose major clinical challenges [1].

Gastric cancer (GC) is a heterogeneous malignancy and remains one of the primary global causes of death from cancer [2]. Its mortality rates and incidence are continuing to rise in several regions, particularly in East Asia, followed by Eastern and Central Europe [3]. Despite recent advancements in early identification and therapy, the general prognosis for GC patients is still dismal, primarily due to late-stage diagnosis and resistance to conventional therapies [4]. Current treatment modalities, including surgery, chemotherapy, and targeted therapies, have shown limited ability to enhance long-term survival results [5]. Recent investigations have shown that chromatin instability and dysregulated chromatin remodeling contribute significantly to gastric cancer progression by altering gene expression, influencing DNA repair, and facilitating oncogenic processes through aberrant epigenetic mechanisms [6,7]. Moreover, DNA damage induced by oxidative stress and environmental carcinogens serves as a major contributor to GC pathogenesis [8]. These findings underscore the significance of clarifying the molecular mechanisms underlying GC development. Given the limited effectiveness of current diagnostic and therapeutic strategies, there is an immediate demand to found new biomarkers and therapeutic targets to improve patient survival.

Cell division cycle associated 7 (*CDCA7*) encodes a protein related to chromatin remodeling and transcriptional regulation [9]. Dysregulation of *CDCA7* impairs the DNA damage repair process, leading to genomic instability [10,11]. Wassing et al. discovered that *CDCA7* recognizes hemimethylated CpGs in the nucleosome core and promotes replication-independent maintenance of DNA methylation by recruiting HELLS [12]. This recruitment maintains DNA methylation and amplifies UHRF1-mediated histone H3 ubiquitination. Furthermore, Vukic et al. highlighted the essential role of *CDCA7* in maintaining global DNA methylation and regulating tissue-specific gene expression [13]. Mutations in *CDCA7* result in widespread DNA hypomethylation, ultimately affecting transcriptional regulation. Notably, Guo et al. reported elevated *CDCA7* expression in GC tissues and proposed that *CDCA7* may modulate inflammatory responses via the TLR4/NF-κB pathway, offering novel insights into its function in GC pathogenesis [14]. These findings collectively reveal that *CDCA7* could be a critical epigenetic regulator in GC, yet its precise molecular mechanisms remain incompletely understood.

Recent studies have indicated the function of epigenetic and genetic variations in GC. *CDCA7* has emerged as a potential candidate gene due to its involvement in regulating DNA methylation, chromatin remodeling, and gene expression. Given its association with genomic instability, *CDCA7* may be important in the pathophysiology of GC. Furthermore, the interaction between *CDCA7* and the chromatin remodeling protein HELLS suggests that these molecules may coordinate key oncogenic processes such as cell proliferation, apoptosis, and invasion. This relationship implies that *CDCA7* may influence GC progression through HELLS-dependent chromatin regulatory mechanisms.

Investigating *CDCA7*’s functional involvement in GC and exploring its capacity as a biomarker and target for early diagnosis and treatment techniques were the goals of this investigation.

## 2 Materials and Methods

### 2.1 Data Sources and Differential Expression Analysis

The ASSISTANT for Clinical Bioinformatics (https://www.aclbi.com/static/index.html#/) was employed to obtain the GC dataset from The Cancer Genome Atlas (TCGA). This dataset contains 375 GC samples and 32 samples of normal tissue. Additionally, two independent GC-related gene expression datasets were selected from the GEO (https://www.ncbi.nlm.nih.gov/gds/). There are 5 GC samples and 5 normal tissue samples in the GSE56807 dataset, while the GSE19826 dataset contains 5 GC samples and 5 normal tissue samples. Differential gene expression analysis was applied on the TCGA-GC, GSE19826, and GSE56807 datasets using the “Limma” package in R software. Genes with |log_2_ Fold Change (FC)| > 1 were considered upregulated differentially expressed genes (DEGs), while genes with |log_2_FC| < 1 were classified as downregulated DEGs. A statistical significance threshold of *p* < 0.05 was applied to identify meaningful DEGs. In the TCGA-GC dataset, the 32 normal tissue samples represent adjacent non-tumorous gastric tissues collected from patients within the TCGA cohort, rather than tissues from independent healthy donors.

### 2.2 Topological and Functional Enrichment Analysis

To conduct topological analysis, which refers to identifying the relationships and interactions between upregulated and downregulated DEGs, the Bioinformatics and Evolutionary Genomics Web Tools (https://bioinformatics.psb.ugent.be/webtools/Venn/) visualizing and analyzing the overlap and distribution of the selected DEGs across the TCGA-GC, GSE19826, and GSE56807 datasets. For functional enrichment analysis of the selected genes, the DAVID tool (version 6.8, released in July 2020, https://david.ncifcrf.gov/tools.jsp) was employed. This comprehensive analysis encompassed the Kyoto Encyclopedia of Genes and Genomes (KEGG) pathway, as well as Gene Ontology (GO) analysis, which covered all three categories: Biological Process, Molecular Function, and Cellular Component.

### 2.3 Protein-Protein Interaction (PPI) Network and Identification of Hub Genes

The PPI network analysis was applied by the STRING database (version 11.5, available from https://string-db.org/). The analysis was conducted for *Homo sapiens* with a confidence threshold of 0.4. The interactions included in the network were experimental, database, co-expression, and text-mining types. Based on the results from the STRING database, only interactions with a confidence score greater than 0.4 were considered in the analysis. To detect key candidate genes, the cytoHubba plugin (version 0.1, The Cytoscape Consortium, San Diego, CA, USA) in Cytoscape (version 3.8.2, The Cytoscape Consortium) was utilized, applying the DMNC and ClusteringCoefficient algorithms. For each algorithm, the top 15 genes with the highest scores were chosen. Subsequently, topological overlap analysis was applied to the selected genes. This method evaluates the similarity between genes based on the number of shared neighbors in the PPI network. Genes with high overlap are considered more functionally related. A threshold of topological overlap >0.1 was applied to filter significant gene pairs. This approach helped identify hub genes that are highly interconnected in the PPI network. The analysis was conducted using the Bioinformatics and Evolutionary Genomics Web Tools (https://bioinformatics.psb.ugent.be/webtools/Venn/).

### 2.4 Receiver Operating Characteristic (ROC) Curve and Expression Analysis

To determine the diagnostic potential of six candidate genes, ROC curves were generated with the R package “timeROC” based on the GSE19826 and GSE56807 datasets. The area under the curve (AUC) was used to detect the discriminatory ability of each gene. In the timeROC analysis, the “timeROC” package was used with the following parameters: timepoint = 3, representing the time point fsurvival analysis (in years); censor = TRUE, indicating that censored data were included; method = “KM”, using Kaplan-Meier estimation for survival curves; and conf.level = 0.95 for calculating the 95% confidence interval (CI) of the AUC. The confidence interval was computed using bootstrap resampling (1000 iterations) to estimate the variability of the AUC. *CDCA7* expression in normal vs. tumor tissues was analyzed utilizing the TCGA-GC dataset (version 2018, Type: RNA-seq and clinical data) via the ASSISTANT for Clinical Bioinformatics platform. Additionally, the Sangerbox platform (http://vip.sangerbox.com/home.html) was used to visualize and compare *CDCA7* levels from the GSE19826 and GSE56807 datasets in both tumor and normal samples.

### 2.5 Cell Lines and Culture Conditions

GC cell lines HGC-27 (Catalog No. SCSP-5263) and AGS (Catalog No. SCSP-5262) and normal gastric mucosal cell GES1 (Catalog No. SCSP-308) were purchased from the Shanghai Cell Bank of the Chinese Academy of Sciences (Shanghai, China). HGC-27 and AGS are derived from a poorly differentiated gastric carcinoma with a p53 mutation and a moderately differentiated gastric adenocarcinoma with a KRAS mutation, respectively. These cell lines possess distinct genetic backgrounds, justifying their combined use for mechanistic validation. The AGS cell line was cultivated in F-12K medium (31765-029, Gibco, Grand Island, NY, USA) containing 10% high-quality fetal bovine serum (FBS, 26140-079, Gibco). GES1 and HGC-27 cells were planted in DMEM (11965-092, Gibco) fortified with 10% FBS (26140-079, Gibco). All cultures were incubated at 37°C in a humidified atmosphere with 5% CO_2_. Cell line authentication was performed using short tandem repeat (STR) profiling, and all cell lines tested negative for mycoplasma contamination prior to experimentation.

### 2.6 Cell Transfection

Cells were transfected with either an overexpression plasmid of *HELLS* (pcDNA3.1-*HELLS*) or the corresponding empty vector control (pcDNA3.1). To achieve gene knockdown, targeting *CDCA7* or a non-targeting control siRNA, small interfering RNAs (siRNAs) were transfected into cells. The siRNA sequence for the non-targeting control (si-NC) was as follows: forward 5^′^-UUCUCCGAACGUGUCACGUTT-3^′^, reverse 5^′^-AAACGUGACACGUUCGGAGAA-3^′^. The siRNA sequence targeting *CDCA7* (si-*CDCA7*) was as follows: forward 5^′^-CCUCUGAUGACAGUUGUGACA-3^′^, reverse 5^′^-UGUCACAACUGUCAUCAGAGG-3^′^. For knockdown of *CDCA7*, **s**iRNA was used at a concentration of 50 nM. With the manufacturer’s protocol, transfection of the specific siRNAs and plasmids was undertaken with Lipofectamine^TM^ 3000 transfection reagent (Invitrogen, Waltham, MA, USA). Cells were taken out for subsequent analysis after a 48 h transfection period. Each experimental condition was performed in triplicates (biological replicates) with three technical replicates per condition. The efficiency of gene knockdown was validated by Western blotting for CDCA7 protein expression. A significant reduction in CDCA7 protein expression was observed in cells transfected with the *CDCA7*-targeting siRNA compared to the control siRNA-transfected cells, confirming successful knockdown.

### 2.7 Quantitative Real-Time PCR (qRT-PCR)

As previously disclosed, total RNA extraction, reverse transcription, and qRT-PCR were conducted [15]. They were normalized to an internal standard, *GAPDH*. The 2^−ΔΔCt^ technique was applied to calculate relative gene expression [16]. qRT-PCR primers included: *CDCA7*: forward 5^′^-GGCCGGTATTTTTCATGCCG-3^′^, reverse 5^′^-AACTTCATCGCCACCCTGAG-3^′^; *HELLS*: forward 5^′^-ACTGGTACTCCCTTGCAAAACA-3^′^, reverse 5^′^-AGGAATTCTTTCCTGGTGCAG-3^′^; and *GAPDH*: forward 5^′^-TCAGCCGCATCTTCTTTTGC-3^′^, reverse 5^′^-ACCTTCCCCATGGTGTCTGA-3^′^. Each experiment was performed with three biological replicates and three technical replicates for each condition. The results were averaged from the technical replicates, and statistical analysis was performed using the mean values from the biological replicates.

### 2.8 Western Blotting (WB)

Cells were lysed in RIPA buffer (P0013B, Beyotime, Shanghai, China) containing protease inhibitors (C0009, CoWin Biosciences, Nanjing, China). Protein levels were determined with the BCA assay (P0012S, Beyotime). Following resolution on 10% SDS-PAGE gels, equal quantities (30 μg) of protein were transferred to PVDF membranes (F0015, Beyotime). The membranes were incubated in 5% non-fat milk prepared for 1 h in TBST at room temperature (RT) and incubated overnight with primary antibodies against CDCA7 (1:1000, 15249-1-AP, Proteintech, Wuhan, China), H3K9me3 (1:2000, ab8898, Abcam, Cambridge, UK), HELLS (1:2000, 11955-1-AP, Proteintech), H4K20me3 (1:1000, ab177190, Abcam), and GAPDH (1:2000, ab8245, Abcam) at 4°C. The membranes were cleaned and then incubated with HRP-labeled Rabbit Anti-Mouse IgG (1:10000, ab6728, Abcam) for 1 h at RT. ImageJ software (version 1.8.0, NIH, Bethesda, MD, USA) was applied to measure the protein bands, which were detected with an ECL detection system (Tiangen, Beijing, China).

### 2.9 Dot Blot Analysis

A NanoDrop spectrophotometer (ND-1000, Thermo Fisher Scientific, Waltham, MA, USA) was used to calculate the quality and amount of genomic DNA (gDNA), which was isolated from cultivated cells via a DNA isolation kit (69504, Qiagen, Hilden, Germany). The starting material for DNA extraction was approximately 1 × 10^6^ cells, and the elution volume was 50 μL. Dot blot experiments were conducted as previously described [17], with minor modifications, including using a lower concentration of primary antibodies (1:500 for 5 hmC and 1:1000 for 5 mC) and reducing the incubation time with the antibodies to 12 h instead of the previously reported 18 h. Membranes were blocked with 5% non-fat milk for 1 h at RT in TBST, followed by overnight incubation with primary antibodies against 5 hmC (1:500, ab106918, Abcam) and 5 mC (1:1000, ab307566, Abcam) at 4°C. After washing, for 1 h at RT, membranes were probed with goat-derived HRP-conjugated antibodies targeting mouse IgG (1:5000, ab6789, Abcam) and rat IgG (1:5000, ab7097, Abcam). Signals were detected utilizing an ECL detection system (Tiangen) and exposed for 30 s to 5 min depending on the signal intensity. Image analysis was performed using ImageJ (version 1.8.0, NIH).

### 2.10 Co-Immunoprecipitation (Co-IP) Assay

To investigate the connection between CDCA7 and HELLS in GC cells, cells were seeded at a density of 1 × 10^6^ cells per 10 cm dish and lysed in ice-cold IP lysis buffer (P0013C, Beyotime) supplied with protease and phosphatase inhibitors. After centrifuging lysates at 12,000× *g* for 15 min, the supernatants were collected. Approximately 500 μg of total protein was incubated overnight with either anti-CDCA7 antibody (1:500, 15249-1-AP, Proteintech) or anti-HELLS antibody (1:500, 11955-1-AP, Proteintech) at 4°C. The mixture was incubated with 50 μL of Protein A/G magnetic beads (88804, Thermo Fisher Scientific) at 4°C for 4 h under gentle rotation. The beads underwent boiling in SDS loading buffer to elute the bound proteins after three rounds of washing with cold lysis buffer. For WB analysis, 50 μL of the eluted sample was loaded per lane, and the protein concentration was normalized using the BCA assay (P0012S, Beyotime) to ensure equal protein loading across lanes. Immunoprecipitates were analyzed by WB using the indicated antibodies.

### 2.11 Chromatin Immunoprecipitation (ChIP) Assay

ChIP assays were carried out to assess the chromatin recruitment of HELLS and CDCA7 following *CDCA7* knockdown. Cells were seeded at a density of 1 × 10^6^ cells per 10 cm dish, transfected with si-*CDCA7* (50 nM) or si-NC (50 nM) using Lipofectamine^TM^ 3000 (Invitrogen, USA) according to the manufacturer’s protocol. After 48 h of culture, cells were cross-linked for 10 min at ambient temperature using 1% formaldehyde. The cross-linking reaction was terminated by adding 125 mM glycine and incubating for 5 min. Cells were harvested and lysed in ChIP lysis buffer (P0012, Beyotime), and chromatin was sonicated into 200–500 bp fragments. Fragmented chromatin was placed overnight at 4°C with anti-HELLS (1:500, 11955-1-AP, Proteintech), anti-CDCA7 (1:500, 15249-1-AP, Proteintech), or IgG control antibodies. Protein A/G used to capture immune complexes, followed by three washes with cold ChIP buffer (P0012, Beyotime) under gentle rotation. After washing, complexes were eluted and reverse-cross-linked for 4 h at 65°C. The eluted protein-DNA complexes were subjected to WB analysis to evaluate the levels of chromatin-bound CDCA7 and HELLS. Three biological replicates and technical duplicates were used to ensure reproducibility of the ChIP experiments.

### 2.12 Colony Formation Assay

A colony formation evaluation was applied to measure cell proliferation. Cells (500 per well) were kept in six-well plates and cultured in DMEM (11965-092, Gibco) supplemented with 10% FBS (26140-079, Gibco). The cells were incubated at 37°C in a humidified atmosphere with 5% CO_2_ for 10 days. Cells were rinsed with PBS to eliminate any remaining medium. Colonies were then fixed with alkali nitro blue tetrazolium chloride solution at RT for 20 min. Following fixation, the colonies were washed again with PBS to remove excess dye and were photographed with an Olympus IX73 microscope (Olympus, Tokyo, Japan). Colonies were counted microscopically, and formation rates were calculated as the number of colonies per well relative to the initial seeding density. Three biological replicates were used for each condition.

### 2.13 Cell Counting Kit-8 (CCK-8) Assay

The CCK-8 assay (KGA217, KeyGEN, Nanjing, China) was performed to determine cell viability. Cells were cultivated in 96-well plates at 5 × 10^3^ cells per well and, following the indicated treatments, CCK-8 solution was added. Optical density at 450 nm was obtained with a microplate reader (KHB-3000, Kehua Technologies, Shanghai, China) on days 0, 1, 2, 3, and 4. Cell viability was calculated using the following formula:

$$Cell\;viability=\frac{OD\;of\;treated\;group-OD\;o\;f\;blank\;group}{OD\;o\;f\;control\;group-OD\;o\;f\;blank\;group}\times 100\text{\% }$$

### 2.14 Cell Migration and Invasion Assays

The ability of transfected cells to invade and migrate was assessed with transwell assays. For the migration assay, 1 × 10^5^ cells were planted into the top chambers of the Transwell insert (3422, Corning, NY, USA) after being suspended in serum-free DMEM (11965-092, Gibco). The bottom chambers were filled with medium that included 10% FBS (26140-079, Gibco). Following 48 h of 37°C incubation, non-migrated cells were gently wiped from the upper side of the membrane. Cells that had traversed to the lower surface were labeled with DAPI (Catalog No. D3571, Thermo Fisher Scientific) at a concentration of 1 μg/mL for 15 min, fixed with 4% paraformaldehyde for 10 min, and examined under an inverted fluorescence microscope (Olympus IX73, Japan; 200×). Five randomly chosen microscopic fields were imaged for counting. Matrigel (Corning, NY, USA) was diluted at a ratio of 1:4 with serum-free DMEM, and 50 μL of the diluted Matrigel was applied to coat the upper chamber membranes. The coated membranes were incubated at 37°C for 1 h to allow polymerization. The remaining steps were carried out as described in the migration assay. Three biological replicates were used for each experimental condition.

### 2.15 Flow Cytometry Analysis

The Annexin V-FITC Apoptosis Detection Kit (E-CK-A314, Elabscience, Wuhan, China) was applied in flow cytometry to investigate cell apoptosis. Following treatment, 1 × 10^6^ cells per sample were harvested, washed, and incubated with 5 μL of Propidium Iodide (50 μg/mL) and 5 μL of Annexin V-FITC (1:20 dilution) in a final volume of 200 μL of binding buffer at room temperature (RT) for 15 min. The apoptotic cell population was processed utilizing a flow cytometer (BD FACSCanto II, BD Biosciences, Franklin Lakes, NJ, USA), with the following settings: 488 nm laser for Annexin V-FITC detection, 635 nm laser for PI detection, and 530/30 nm filter for Annexin V-FITC, 585/42 nm filter for PI. Data from 10,000 events per sample were collected, and the apoptotic population was analyzed using FlowJo software (version 10.8.1, FlowJo LLC, Ashland, OR, USA).

### 2.16 Statistical Analysis

All statistical procedures were executed in R (version 4.2.2). Data from triplicate independent studies are shown as the mean ± standard deviation (SD). The Wilcoxon rank-sum test or Student’s *t*-test was employed to compare two groups. For multiple group comparisons, ANOVA followed by Tukey’s post hoc adjustment was conducted. Results with *p* < 0.05 were regarded as statistically significant.

## 3 Results

### 3.1 Differentially Expressed Genes and Functional Enrichment Analysis in GC

**Figure 1:**
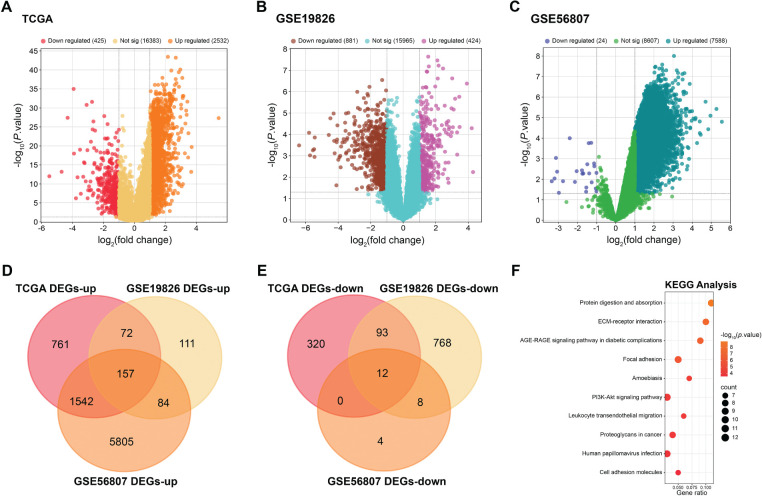

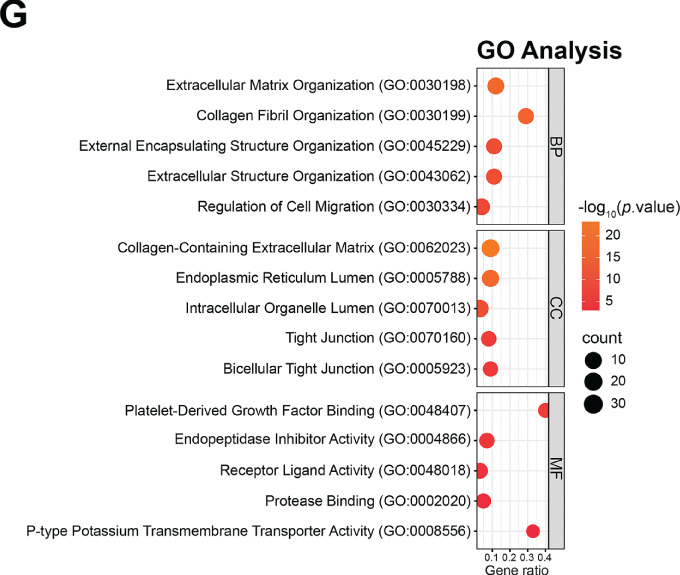
Differential expression analysis and functional enrichment of gastric cancer-related genes. (**A**) Volcano plots showing differentially expressed genes (DEGs) in gastric cancer from the Cancer Genome Atlas (TCGA) datasets. |log_2_ Fold Change (FC)| > 1. (**B**) Volcano plots showing DEGs in gastric cancer from the GSE19826 datasets. |log_2_FC| > 1. (**C**) Volcano plots showing DEGs in gastric cancer from the GSE56807 datasets. |log_2_FC| > 1. (**D**) Venn diagrams of overlapping upregulated DEGs from the TCGA, GSE19826, and GSE56807 datasets. (**E**) Venn diagrams of overlapping downregulated DEGs from the TCGA, GSE19826, and GSE56807 datasets. (**F**) Kyoto Encyclopedia of Genes and Genomes (KEGG) pathway enrichment analysis of the overlapping genes from the three datasets. Dot size indicates gene number, and color indicates −log_10_ (*p* value). (**G**) Gene Ontology (GO) enrichment analysis of the overlapping genes from the three datasets in the biological process (BP), cellular component (CC), and molecular function (MF) categories.

Three GC-related datasets were evaluated by differential expression analysis in this investigation. From the TCGA-GC dataset, 2532 upregulated and 425 downregulated DEGs were identified. In the GSE19826 dataset, 424 upregulated and 881 downregulated DEGs were detected, while in the GSE56807 dataset, 7588 upregulated and 24 downregulated DEGs were observed (Fig. 1A–C). Topological analysis of the upregulated and downregulated DEGs from the three datasets revealed 157 overlapping upregulated DEGs and 12 overlapping downregulated DEGs (Fig. 1D,E). Further functional enrichment analysis of 169 overlapping DEGs was applied. As shown in Fig. 1F, the KEGG pathway analysis emphasized enrichment in the “Cell adhesion molecules”, “Proteoglycans in cancer”, and “ECM-receptor interaction” pathways. Further GO analysis disclosed that the intersecting genes were enriched in “extracellular matrix organization” (BP), “Intracellular Organelle Lumen” (CC), and “Protease Binding” (MF), etc. (Fig. 1G).

### 3.2 CDCA7 is a Highly Expressed Hub Gene with Diagnostic Potential in GC

Previous TCGA-based exploratory studies have shown that large-scale data-driven analyses often highlight chromatin regulators and cell-cycle–related genes as recurrent oncogenic drivers [18]. This background supports the robustness of TCGA-guided discovery and provides a clear rationale for prioritizing genes that repeatedly emerge as cell-cycle–associated candidates across datasets.

**Figure 2:**
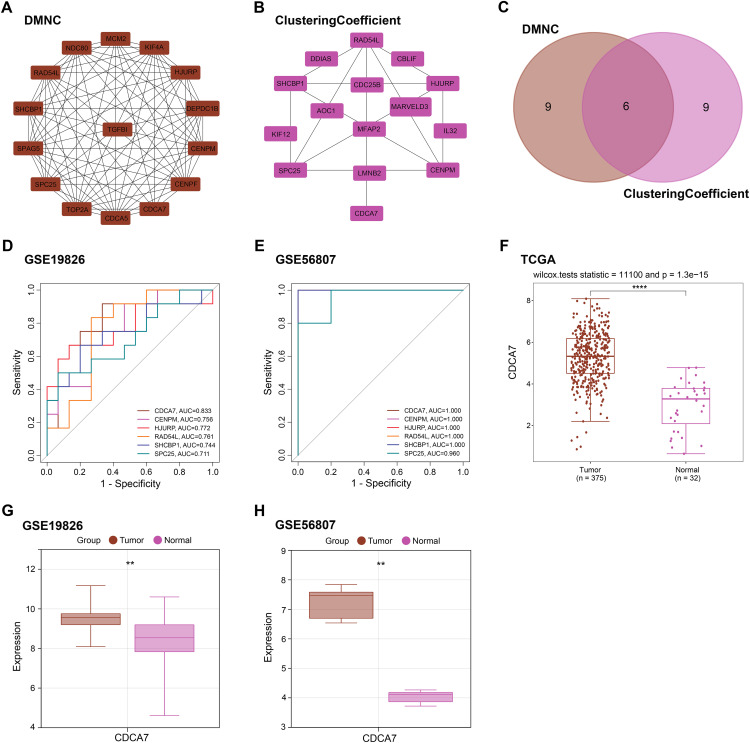
Protein-protein interaction (PPI) network, receiver operating characteristic (ROC) curve, and expression profile analysis of hub genes in gastric cancer. (**A**) Protein-protein interaction (PPI) network analysis of 169 overlapping genes using the Density of Maximum Neighborhood Component (DMNC) topology algorithms. (**B**) PPI network analysis of 169 overlapping genes using the Density of ClusteringCoefficient topology algorithms. (**C**) A Venn diagram shows the overlap of hub genes identified by the DMNC and ClusteringCoefficient algorithms. Six overlapping genes were identified. (**D**,**E**) ROC curve analysis of six candidate hub genes in the GSE19826 (**D**) and GSE56807 (**E**) datasets. *CDCA7* demonstrated the highest diagnostic performance in both datasets (Area under the curves [AUCs] of 0.833 and 1.000, respectively). (**F**) Boxplots of the Cancer Genome Atlas (TCGA) datasets show the expression levels of *CDCA7* in tumor and normal tissues. (**G**) Boxplots of the GSE19826 datasets show the expression levels of *CDCA7* in tumor and normal tissues. (**H**) Boxplots of the GSE56807 datasets show the expression levels of *CDCA7* in tumor and normal tissues. **p < 0.01, ****p < 0.0001.

To investigate the relationships among the overlapping genes, PPI network analysis was applied. With the DMNC and ClusteringCoefficient algorithms, the top 15 top genes were selected (Fig. 2A,B). Venn analysis was then performed to intersect the gene sets obtained from both algorithms, resulting in six candidate genes (*CDCA7*, *CENPM*, *HJURP*, *RAD54L*, *SHCBP1*, *SPC25*) (Fig. 2C). To evaluate the potential of these candidate genes as biomarkers for GC, ROC curve analysis was conducted using the GSE19826 and GSE56807 datasets. In the GSE19826 dataset, *CDCA7* exhibited the best predictive performance (AUC = 0.833, Fig. 2D). In the GSE56807 dataset, *CDCA7*, *CENPM*, *HJURP*, *RAD54L*, and *SHCBP1* all demonstrated excellent predictive accuracy (AUC = 1, Fig. 2E). Given the high predictive performance of *CDCA7* and the relatively limited research on its role in GC, *CDCA7* was designated as the hub gene for follow-up investigation. Expression analysis disclosed that *CDCA7* was upregulated in tumors from the TCGA-GC, GSE19826, and GSE56807 datasets, suggesting a potential tumor-promoting role in GC (Fig. 2F–H).

### 3.3 CDCA7 Knockdown Suppresses Proliferation and Induces Apoptosis in GC Cells

**Figure 3:**
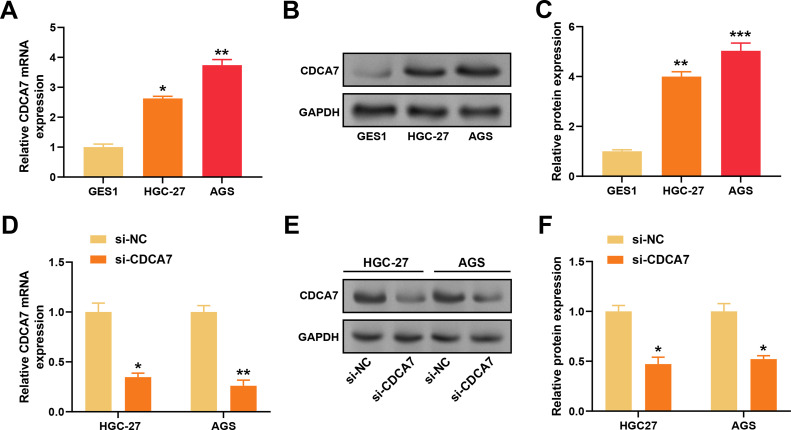
*CDCA7* is highly expressed in gastric cancer cell lines and effectively silenced by siRNA. (**A**) Quantitative real-time polymerase chain reaction (qRT-PCR) analysis of *CDCA7* mRNA expression in normal gastric epithelial cells (GES1) and gastric cancer cell lines (HGC-27 and AGS). (**B**,**C**) Western blotting analysis of *CDCA7* protein expression in normal gastric epithelial cells (GES1) and gastric cancer cell lines (HGC-27 and AGS). (**D**) qRT-PCR analysis of *CDCA7* knockdown efficiency in HGC-27 and AGS cells transfected with si-*CDCA7* or negative control siRNA (si-NC). (**E**,**F**) Western blotting (**E**) and quantitative analysis (**F**) showed that CDCA7 protein levels were reduced in HGC-27 and AGS cells after si-*CDCA7* transfection. n = 3 independent experiments, *p < 0.05, **p < 0.01, ***p < 0.001.

**Figure 4:**
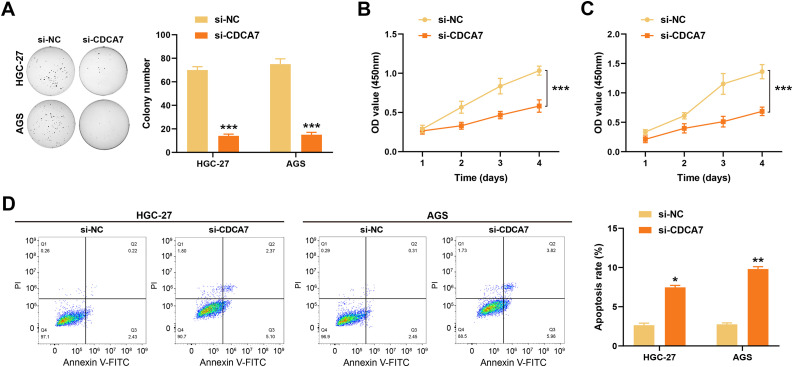
*CDCA7* knockdown inhibits gastric cancer cell proliferation and promotes apoptosis. (**A**) Colony assay to assess the clonogenicity of HGC-27 and AGS cells after *CDCA7* silencing. (**B**,**C**) Cell counting kit-8 (CCK-8) assay to assess cell proliferation in HGC-27 and AGS cells. (**D**) Flow cytometry analysis of apoptosis in HGC-27 and AGS cells using Annexin V-FITC/Propidium Iodide (PI) staining. The bar graph shows that *CDCA7* silencing significantly increased the rate of cell apoptosis compared to the si-NC group. n = 3 independent experiments, *p < 0.05, **p < 0.01, ***p < 0.001.

To assess the *CDCA7* expression, qRT-PCR and WB analyses were performed on normal gastric cells (GES1) and GC cells (HGC-27 and AGS). The results demonstrated significantly higher expression of *CDCA7* in the HGC-27 and AGS cells compared to the normal GES1 cells (Fig. 3A–C). CDCA7 knockdown efficiency in GC cells was confirmed by qRT-PCR and WB analyses. With the control group, *CDCA7* knockdown caused a substantial reduction in both *CDCA7* mRNA and protein expression (Fig. 3D–F). To examine the functional impact of *CDCA7* depletion on cell proliferation, colony formation assays and CCK-8 assays were conducted. The findings revealed that after *CDCA7* knockdown, cell proliferation decreased (Fig. 4A–C). Additionally, flow cytometry analysis showed an increased rate of apoptosis in *CDCA7*-silenced cells relative to controls (Fig. 4D).

### 3.4 CDCA7 Facilitates HELLS Chromatin Recruitment and Physically Interacts with HELLS

**Figure 5:**
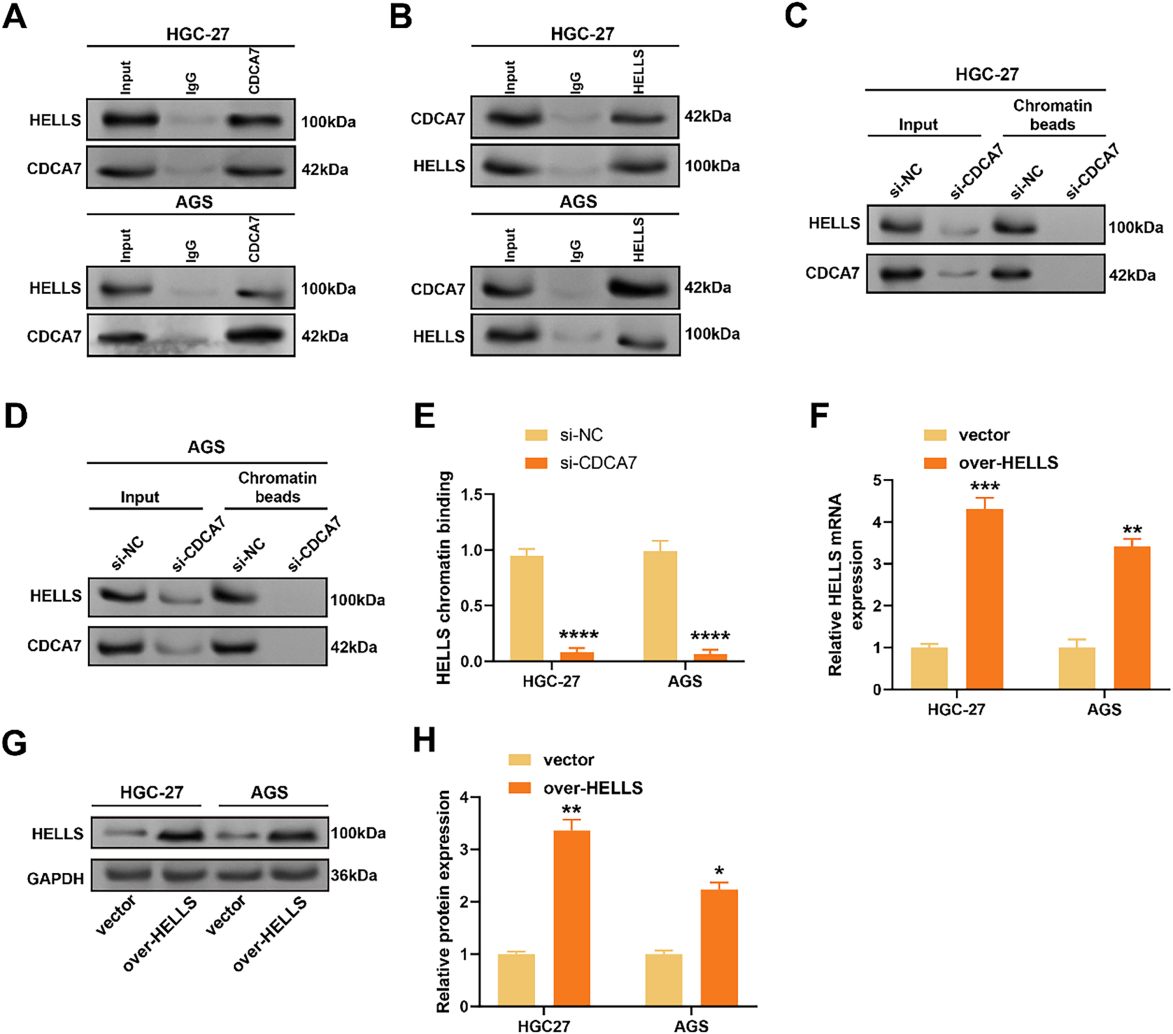
*CDCA7* promotes chromatin recruitment of HELLS in gastric cancer cells. (**A**,**B**) Co-immunoprecipitation (Co-IP) experiments confirmed the interaction between CDCA7 and HELLS proteins in HGC-27 and AGS cells. (**A**) Immunoprecipitation with anti-CDCA7 antibodies downregulates HELLS; (**B**) Reverse Co-IP experiments with anti-HELLS antibodies confirmed the interaction. (**C**–**E**) Chromatin immunoprecipitation (ChIP) experiments analyzed the recruitment of HELLS to chromatin after *CDCA7* knockdown. *CDCA7* silencing (si-*CDCA7*) reduced the level of chromatin-bound HELLS in HGC-27 and AGS cells. (**F**) qRT-PCR analysis of the transfection efficiency of *HELLS* overexpression in HGC-27 and AGS cells. (**G**,**H**) Western blotting (**G**) and quantitative analysis (**H**) showed that HELLS protein levels were significantly increased in HGC-27 and AGS cells after *HELLS* overexpression. n = 3 independent experiments, *p < 0.05, **p < 0.01, ***p < 0.001, ****p < 0.0001.

Previous studies have suggested that CDCA7 can guide HELLS to target specific DNA regions, assisting in DNA methylation and chromatin remodeling [19,20]. To clarify the potential regulatory association between CDCA7 and HELLS in GC cells, Co-IP assays were performed. CDCA7 was shown to physically interact with HELLS in GC cells (Fig. 5A), and reciprocal Co-IP confirmed this interaction (Fig. 5B). To further assess whether CDCA7 affects HELLS chromatin recruitment, a ChIP assay followed by western blotting was conducted. Knockdown of *CDCA7* markedly reduced HELLS binding to chromatin in GC cells (Fig. 5C–E), indicating that CDCA7 facilitates HELLS recruitment to chromatin. Additionally, *HELLS* levels were clearly elevated in GC cells transfected with over-*HELLS* plasmids in comparison to controls, as demonstrated by qRT-PCR and WB analyses (Fig. 5F–H).

### 3.5 Modulation of DNA Methylation and Chromatin Stability Proteins by CDCA7 and HELLS in GC Cells

**Figure 6:**
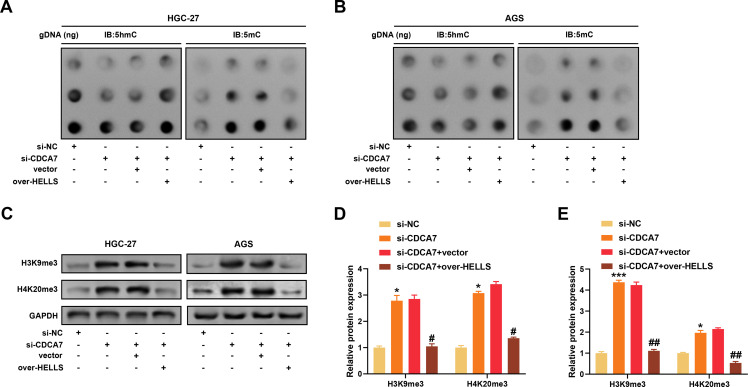
*CDCA7* regulates DNA methylation and histone trimethylation in gastric cancer cells via *HELLS*. (**A**,**B**) Dot blot assays were performed to detect levels of 5-hydroxymethylcytosine (5 hmC) and 5-methylcytosine (5 mC) in genomic DNA isolated from HGC-27 (**A**) and AGS (**B**) cells under different treatment conditions. *CDCA7* knockdown reduced global 5 mC and 5 hmC levels, while overexpression of *HELLS* partially restored them. (**C**) Western blotting analysis of heterochromatic histone trimethylation markers H3K9me3 and H4K20me3 in HGC-27 and AGS cells under *CDCA7* silencing and/or *HELLS* overexpression. (**D**,**E**) Quantification of H3K9me3 and H4K20me3 protein expression from panel C in (**D**) HGC-27 and (**E**) AGS cells. *CDCA7* knockdown significantly increased these histone marks, and *HELLS* overexpression partially rescued the effect. n = 3 independent experiments, *p < 0.05, ***p < 0.001 vs. si-NC group; ^#^*p* < 0.05, ^##^*p* < 0.01 vs. si-*CDCA7*+vector.

Aiming to uncover the epigenetic regulatory function of *CDCA7* and *HELLS* in GC, dot blot assays were performed to assess global DNA methylation levels. As shown in Fig. 6A,B, knockdown of *CDCA7* reduced the levels of 5 hmC and 5 mC in both AGS and HGC-27 cells, whereas overexpression of *HELLS* partially restored their levels. To investigate chromatin stability, western blotting was conducted to detect histone methylation markers H3K9me3 and H4K20me3. *CDCA7* silencing significantly increased the levels of both histone marks, while *HELLS* overexpression partially reversed these increases (Fig. 6C). Quantitative analysis further supported these observations (Fig. 6D,E). Collectively, these outcomes imply that *CDCA7* and *HELLS* work together to preserve DNA methylation and chromatin stability in GC cells.

### 3.6 CDCA7 Knockdown Suppresses Malignant Phenotypes of GC Cells, Partially Reversed by HELLS Overexpression

**Figure 7:**
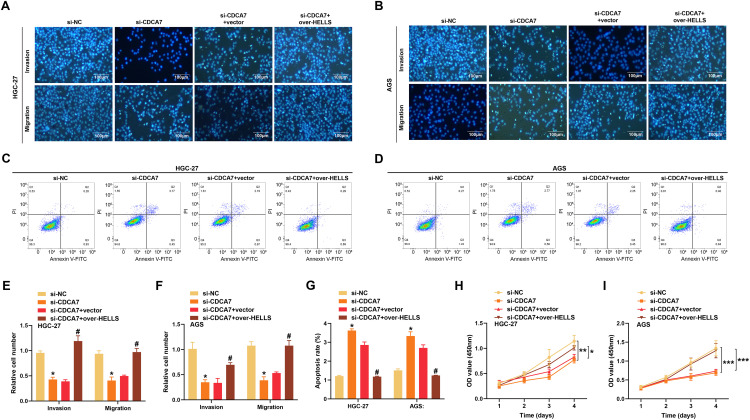
*HELLS* overexpression reverses *CDCA7* knockdown-mediated suppression of proliferation, invasion, and migration while reducing apoptosis in gastric cancer cells. (**A**) Representative images of transwell invasion and migration assays examining changes in cell invasion and migration following *CDCA7* knockdown alone or in combination with *HELLS* overexpression in HGC-27 cells. Scale bar = 100 μm. (**B**) Representative images of transwell invasion and migration assays examining changes in cell invasion and migration following *CDCA7* knockdown alone or in combination with *HELLS* overexpression in AGS cells. Scale bar = 100 μm. (**C**) Apoptosis in HGC-27 cells was assessed by flow cytometry using Annexin V-FITC/Propidium Iodide (PI) staining. (**D**) Apoptosis in AGS cells was assessed by flow cytometry using Annexin V-FITC/PI staining. (**E**) Quantification of invasion and migration of HGC-27 cells using transwell assays. *p < 0.05 vs. si-NC group; ^#^*p* < 0.05 vs. si-*CDCA7*+vector group. (**F**) Quantification of invasion and migration of AGS (**F**) cells using transwell assays. *p < 0.05 vs. si-NC group; ^#^*p* < 0.05 vs. si-*CDCA7*+vector group. (**G**) Quantification of apoptosis in HGC-27 and AGS cell lines using flow cytometry data. *p < 0.05 vs. si-NC group; ^#^*p* < 0.05 vs. si-*CDCA7*+vector group. (**H**,**I**) Cell counting kit-8 (CCK-8) assays assessing proliferation of HGC-27 (**H**) and AGS (**I**) cells following *CDCA7* knockdown alone or combined with *HELLS* overexpression. n = 3 independent experiments, *p < 0.05, **p < 0.01, ***p < 0.001.

To further assess the impacts of *CDCA7* and *HELLS* on GC cell behaviors, Transwell assays were applied to evaluate invasion and migration of cells following transfection with si-*CDCA7* and over-*HELLS* in GC cell lines. Compared to the control group, si-*CDCA7* inhibited both invasion and migration of HGC-27 and AGS cells. The inhibition of cell migration and invasion caused by si-*CDCA7* was reversed by overexpression of *HELLS* (Fig. 7A,B,E,F). Flow cytometry analysis revealed that si-*CDCA7* stimulated apoptosis in both HGC-27 and AGS cells, while overexpression of *HELLS* effectively countered this pro-apoptotic effect (Fig. 7C,D,G). Additionally, CCK-8 assays showed that si-*CDCA7* reduced cell proliferation in HGC-27 and AGS cells, and overexpression of *HELLS* restored the inhibited proliferation (Fig. 7H,I).

## 4 Discussion

This study establishes *CDCA7* as a novel oncogenic hub in GC through integrated multi-omics analysis and functional assessment. We discovered *CDCA7* as a consistently overexpressed gene across multiple GC cohorts with superior diagnostic potential. *In vitro* experiments proved that *CDCA7* knockdown suppressed GC cell colony formation and proliferation while inducing apoptosis. Mechanistically, we revealed a previously unreported interaction between *CDCA7* and *HELLS* in GC cells. Critically, *CDCA7* depletion reduced DNA methylation marks (5 mC/5 hmC), whereas *HELLS* overexpression reversed *CDCA7* knockdown effects on chromatin stability markers (H3K9me3/H4K20me3). *CDCA7* was found to interact with *HELLS*, a protein involved in chromatin remodeling. *HELLS* overexpression reversed the impacts of *CDCA7* knockdown on invasion, migration, and proliferation. These results suggest that *CDCA7*, through its interaction with *HELLS*, is important for the development of GC.

In advanced stages, GC has a terrible prognosis and is still one of the most common malignancies [21]. Consequently, numerous studies have focused on the diagnosis and treatment of GC. Previous research, such as that by Matsuoka and Yashiro highlighted the application of bioinformatics in identifying GC biomarkers, emphasizing the use of large-scale data and computational methods to uncover key molecular targets for targeted therapies [22]. Differential expression analysis, functional enrichment, and PPI network analyses were employed to detect six candidate genes (*CDCA7*, *CENPM*, *HJURP*, *RAD54L*, *SHCBP1*, *SPC25*) associated with GC progression in our research. It is also noteworthy that contemporary approaches, including generative AI models, are increasingly applied to transcriptome interpretation [23]. While our study did not employ such models, our screening pipeline aligns with this evolving methodological context, illustrating the potential for synthetic data augmentation and more integrative analyses in future research. Among these genes, several were reported that involved in the progression and development of GC. Notably, studies by Li et al. revealed that *HJURP* level is elevated in GC tissues, and silencing *HJURP* significantly suppresses GC cell growth and chemoresistance [24]. Similarly, Pan et al. identified *RAD54L* as a highly expressed gene in GC, correlating with poor prognosis; silencing *RAD54L* promoted cell apoptosis and inhibited proliferation, while its overexpression had the opposite effect [25]. Zhang et al. demonstrated that ginsenoside Rh7 hinders the invasion, migration, and proliferation of GC cells by downregulating *SHCBP1* and inhibiting epithelial-mesenchymal transition (EMT) [26]. These studies support the key roles of the candidate genes we screened in GC development.

Further investigation assessed the clinical diagnostic value of the six candidate genes in GC using ROC curve analysis. Among them, *CDCA7* exhibited the highest AUC value and was therefore selected as the hub gene for subsequent experimental validation. Previous studies have highlighted the association of *CDCA7* with various types of cancer. For example, in esophageal squamous cell carcinoma (ESCC), Li et al. reported that *CDCA7* promotes tumor progression by upregulating *CCNA2* [27]. *CCNA2* expression was positively correlated with *CDCA7*, and its silencing reversed *CDCA7*-induced proliferation and colony formation. Similarly, research by Cai et al. revealed that *CDCA7* is highly expressed in ovarian cancer tissues and cells [28]. They found that reducing *CDCA7* expression suppresses ovarian cancer cell migration, proliferation, angiogenesis, and invasion, leading to cell cycle arrest. To validate these bioinformatic findings, we performed *in vitro* studies aimed at elucidating the biological function of *CDCA7* in GC cells. The findings demonstrated that, in contrast to normal gastric cells, GC cells have high levels of *CDCA7* expression. Silencing *CDCA7* effectively reduced its mRNA and protein levels, inhibited GC cell colony formation and proliferation, and promoted cell apoptosis. These outcomes support that *CDCA7* is an oncogenic factor in GC and might be a target for treatment.

*HELLS*, a member of the DNA helicases SNF2 family, is essential in DNA repair, chromatin remodeling, and the maintenance of genomic stability [29]. Peixoto et al. reviewed *HELLS* as a key epigenetic regulator responsible for preserving DNA methylation patterns, particularly in pericentromeric heterochromatin [30]. Mutations in *HELLS* have been linked to centromeric instability, immunodeficiency, and facial anomalies syndrome. Additionally, *HELLS* has been implicated in various cancers. For example, silencing *HELLS* was discovered to inhibit the proliferation of pancreatic cancer cells and arrest cell cycle progression [31]. In liver cancer, *HELLS* regulates *MIEF1* to promote tumor growth, and disruption of the *HELLS/MIEF1* axis induces mitochondrial dysfunction and cellular senescence [32]. Bioinformatic analyses further indicate that elevated *HELLS* level is correlated with poor overall survival in several malignancies. A recent study has shown that *CDCA7* can interact with *HELLS* and guide it to hemi-methylated DNA regions to promote DNA methylation and chromatin remodeling [33]. In our study, Co-IP and ChIP assays demonstrated that CDCA7 physically interacts with HELLS and facilitates its recruitment to chromatin in GC cells. *CDCA7* knockdown significantly reduced HELLS chromatin binding, supporting its role as a key recruiter of HELLS in the epigenetic machinery.

Chromatin instability is closely associated with cancer progression and is often characterized by drug resistance, enhanced invasiveness, and high proliferative capacity [34]. One prominent example is the chromosomal instability (CIN) subtype of GC, described by Nemtsova et al., which is typified by chromosomal rearrangements, aneuploidy, and TP53 mutations [35]. CIN is associated with poor prognosis but may respond favorably to adjuvant chemotherapy. At the molecular level, DNA methylation and histone modifications are critical for maintaining chromatin stability [36]. 5 mC typically represses transcription by recruiting repressive complexes, while its oxidized form 5 hmC, generated by TET enzymes, is associated with active demethylation processes [37]. Histone modifications such as H3K9me3 and H4K20me3 are essential for heterochromatin formation and genome integrity [38]. In our study, *CDCA7* silencing led to a reduction in global 5 mC and 5 hmC levels, as revealed by dot blot analysis. In parallel, western blotting showed that H3K9me3 and H4K20me3 levels were also reduced following *CDCA7* knockdown. Importantly, overexpression of *HELLS* partially rescued these epigenetic alterations. Notably, while both DNA methylation (5 mC/5 hmC) and histone methylation marks (H3K9me3/H4K20me3) were affected by *CDCA7* knockdown and *HELLS* overexpression, these represent distinct epigenetic layers: DNA methylation primarily modulates transcriptional repression at the DNA level, whereas histone methylation regulates chromatin compaction and nucleosome dynamics. *HELLS*, as a multifunctional chromatin remodeler, may impact both layers through overlapping yet mechanistically separate pathways. This distinction clarifies that the *CDCA7/HELLS* axis does not act via a single uniform regulatory signal, but coordinates multiple epigenetic mechanisms to maintain chromatin stability and support GC cell proliferation and survival. Functionally, *CDCA7* knockdown significantly suppressed GC cell migration, invasion, and proliferation, while promoting apoptosis. Conversely, *HELLS* overexpression effectively reversed these phenotypes, restoring malignant behavior and reducing apoptotic rates. These results collectively emphasize the crucial part of the *CDCA7/HELLS* axis in coordinating epigenetic regulation and tumor cell behavior in GC.

Although this study offers important insights into the roles of *CDCA7* and *HELLS* in GC, several limitations must be acknowledged. First, the bioinformatics analysis was based on publicly available datasets, potentially subject to inherent biases. The sample sizes in the GSE56807 and GSE19826 datasets are limited, with only 5 GC and 5 normal tissue samples in each, which may impact the generalizability of the findings. Validation in larger, independent clinical cohorts is still required. Second, while *in vitro* experiments have highlighted the functional significance of *CDCA7* and *HELLS* in regulating GC cell growth and apoptosis, these findings have not been validated *in vivo*. The lack of animal model validation and clinical relevance limits their potential for direct therapeutic applications. Future studies are planned to investigate the role of the *CDCA7/HELLS* axis *in vivo*. Third, gastric cancer biology is tightly influenced by the TME, which may modulate *CDCA7/HELLS* signaling. *In vitro* cell line models may not fully capture these effects, such as extracellular vesicle-mediated transfer of metabolites, nucleic acids, or chromatin regulators that participate in epigenetic remodeling. Fourth, although knockdown and Co-IP experiments demonstrated clear interactions between *CDCA7* and *HELLS*, further validation using independent antibodies or additional control inputs would strengthen the reliability of these findings. Moreover, methylation and histone modification data are consistent *in vitro*, but the absence of *in vivo* validation is a limitation. More rigorous experimental designs, such as gradient *HELLS* expression or catalytic mutants, could be employed in future studies to dissect the role of *HELLS* enzymatic activity. Furthermore, the specific domains mediating the *CDCA7/HELLS* interaction and the downstream target genes regulated by *HELLS* in the context of *CDCA7* remain unclear. Further investigation is required to fully elucidate the molecular mechanisms of the *CDCA7/HELLS* axis in epigenetic regulation.

Our data suggest that the *CDCA7*/*HELLS* axis constitutes a therapeutically tractable epigenetic dependency in GC; however, translational evidence remains preliminary. Although targeting epigenetic axes like the *CDCA7/HELLS* pathway is conceptually attractive, clinical translation remains preliminary. Emerging perspectives in cancer therapy indicate that while epigenetic modulators show preclinical promise, their routine clinical use is still limited and requires careful assessment [39]. This perspective tempers our enthusiasm and highlights the need for further rigorous studies before considering clinical applications. To our knowledge, selective, probe-grade pharmacologic or RNA-based modulators that directly target *CDCA7* or the *CDCA7*/*HELLS* interface have not yet been reported, and public pharmaceutical/chemical-biology resources currently provide limited leads. As near-term, hypothesis-generating tests, epigenetic pathway inhibitors that reduce 5 mC or H3K9/H4K20 methylation (e.g., DNMT or histone-methyltransferase inhibitors) could be evaluated *in vitro* to determine whether they recapitulate *CDCA7*-loss phenotypes and whether enforced *HELLS* expression mitigates drug responses, thereby linking genetic dependency to pharmacologic sensitivity. These steps will be essential to determine whether the *CDCA7*/*HELLS* axis is truly druggable in clinically relevant settings.

## 5 Conclusion

This investigation revealed *CDCA7* as a hub gene with strong diagnostic performance in GC and demonstrated its oncogenic role in promoting cell growth while inhibiting apoptosis. Mechanistically, *CDCA7* physically interacts with HELLS and facilitates its recruitment to chromatin, thereby enhancing DNA methylation and chromatin stability through the regulation of epigenetic markers such as 5 mC, 5 hmC, H3K9me3, and H4K20me3. Notably, *HELLS* overexpression partially reversed the suppressive impacts of *CDCA7* knockdown on malignant phenotypes in GC cells. These outcomes highlight the *CDCA7/HELLS* axis as a possible target for therapeutic intervention and an essential epigenetic regulator of GC progression.

## Data Availability

The data that support the findings of this study are available from the Corresponding Author, [Huanqing Li, Li Feng], upon reasonable request.
